# Randomized Phase II JANUS Study of Atacicept in Patients With IgA Nephropathy and Persistent Proteinuria

**DOI:** 10.1016/j.ekir.2022.05.017

**Published:** 2022-05-26

**Authors:** Jonathan Barratt, James Tumlin, Yusuke Suzuki, Amy Kao, Aida Aydemir, Kishore Pudota, Hulin Jin, Hans Gühring, Gerald Appel, JANUS study investigators

**Affiliations:** 1 Department of Cardiovascular Sciences, University of Leicester, Leicester, UK; 2 Emory University School of Medicine, Atlanta, Georgia, USA; 3 Faculty of Medicine, Juntendo University Tokyo, Tokyo, Japan; 4 Global Clinical Development, EMD Serono, Billerica, Massachusetts, USA; 5 Global Biostatistics, EMD Serono, Billerica, Massachusetts, USA; 6 Translational Medicine, the healthcare business of Merck KGaA, Darmstadt, Germany; 7 Global Patient Safety, Merck KGaA, Darmstadt, Germany; 8 Columbia University Medical Center, New York, New York, USA

**Keywords:** APRIL/BLyS inhibitor, atacicept, galactose-deficient IgA1, IgA nephropathy, phase II, proteinuria

## Abstract

**Introduction:**

Patients with IgA nephropathy (IgAN) and persistent proteinuria are at risk of progression to kidney failure. Atacicept is a novel B-cell–targeted immunomodulator, shown to reduce immunoglobulin levels in patients with autoimmune diseases.

**Methods:**

JANUS (NCT02808429) was a phase II study that assessed the safety, pharmacodynamic effects, and efficacy of atacicept in patients with IgAN and proteinuria ≥1 g/d or 0.75 mg/mg on 24-hour UPCR despite maximal standard of care therapy.

**Results:**

A total of 16 patients were randomized 1:1:1 to placebo (*n =* 5), atacicept 25 mg (*n =* 6), or atacicept 75 mg (*n =* 5) once weekly using subcutaneous injection. Twelve (75%) completed ≥48 weeks of treatment; 8 (50%) completed 72 weeks of treatment and the 24-week safety follow-up period. Fourteen patients reported treatment-emergent adverse events (TEAEs). Most TEAEs were mild or moderate in severity. Three patients (placebo *n =* 1; atacicept 25 mg *n =* 2) reported serious TEAEs, none of which were treatment related. Dose-dependent reductions in IgA, IgG, IgM, and galactose-deficient (Gd)-IgA1 with atacicept at week 24 were maintained to week 72. Early reduction in proteinuria was observed at week 24 with atacicept. Renal function progressively declined with placebo but remained stable under exposure to atacicept.

**Conclusion:**

Atacicept has an acceptable safety profile in patients with IgAN and is effective at reducing the levels of pathogenic factor Gd-IgA1, with potential improvements in proteinuria and renal function.

## Graphical abstract

**Figure:**
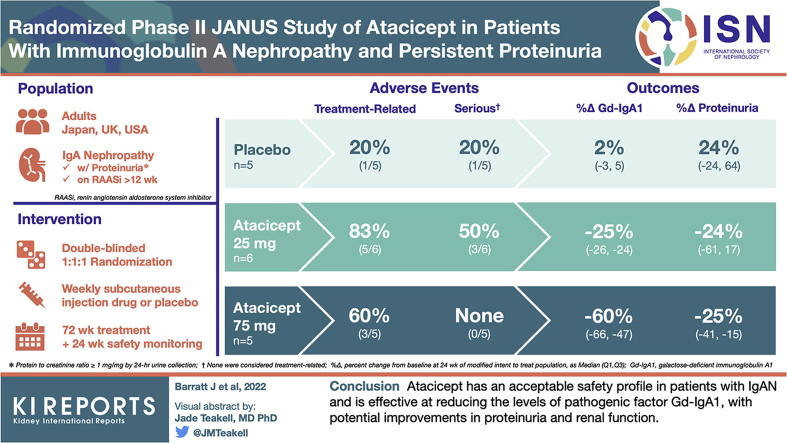

IgAN is the most common primary glomerulonephritis globally.^1^, ^2^, ^3^, ^4^, ^5^, ^6^ Up to 50% of patients with IgAN progress to end-stage renal disease (ESRD) within 20 years, requiring dialysis or kidney transplant, and life expectancy is reduced by 10 years for patients with IgAN.^6^, ^7^, ^8^, ^9^ Persistent proteinuria (urine protein-to-creatinine ratio [UPCR >1 mg/mg]) is the most widely recognized risk factor for renal failure.^3^^,^^10^

Diagnosis of IgAN is based on kidney biopsy revealing IgA1 deposits, which codistribute with complement component C3 in 90% of patients and may also contain IgG or IgM.^6^^,^^11^ Although the pathogenesis of IgAN remains unclear, our understanding of the complex underlying autoimmune mechanisms involved have advanced in recent years.^12^^,^^13^

In response to mucosal stimulation, naïve B cells are activated by cytokines, such as B lymphocyte stimulator (BLyS) and a proliferation-inducing ligand (APRIL), which promote class-switching to IgA antibody-secreting plasma cells that produce Gd and/or polymeric IgA1.^11^^,^^14^ Gd-IgA1 in the circulation may result in the formation of glycan-specific IgA and IgG autoantibodies and IgA1 immune complexes.^14^ These immune complexes are deposited in the glomerular mesangium of the kidney where they cause IgAN-associated kidney injury through several mechanisms.^14^

Mesangial IgA1-immune complex deposition results in the release of proinflammatory and profibrotic mediators leading to mesangial cell proliferation, inflammatory cell recruitment into glomeruli, and an uncontrolled inflammatory response.^11^ Mesangial activation is further amplified by the complement system.^6^ These processes lead to podocyte injury and glomerulosclerosis, as well as proximal tubular epithelial cell activation and tubulointerstitial fibrosis.^11^

There are currently no approved specific pharmacologic treatments for IgAN.^15^ The standard of care is supportive therapy, including angiotensin-converting enzyme inhibitors (ACEi) and angiotensin II receptor blockers (ARBs). These treatments are nonspecific, affect a broad range of immune and inflammatory processes, and only delay rather than halt or reverse the progression to renal failure.^11^^,^^12^ New and specifically targeted treatments are needed.

Atacicept is a human recombinant fusion protein of transmembrane activator and calcium-modulating and cyclophilin ligand interactor and IgG1, under investigation for the treatment of autoimmune diseases.^16^ Atacicept binds to and inhibits BLyS and APRIL, leading to a decrease in B-cell numbers, and interfering with B-cell maturation, differentiation, and effector functions.^16^^,^^17^ Atacicept reduced serum Ig levels in patients with autoimmune diseases,^18^, ^19^, ^20^, ^21^ eliciting a clinical response in a phase IIb study of atacicept in systemic lupus erythematosus with an acceptable safety profile.^21^^,^^22^

Gd-IgA1 serum levels correlate with greater severity of IgAN disease, which suggests that decreasing Gd-IgA1 may slow disease progression.^23^ Nevertheless, despite depleting B cells, the monoclonal antibody rituximab had no effect on serum levels of Gd-IgA1 or anti–Gd-IgA1 in patients with IgAN.^24^ It is postulated that rituximab-resistant mucosal B cells may contribute to the failure of rituximab to reduce Gd-IgA1.^25^^,^^26^ Due to the importance of the mucosal immune system in IgAN, and the critical role of BLyS and APRIL in mucosal B-cell induction, agents targeting the BLyS/APRIL axis offer an alternative therapeutic approach.^11^^,^^25^ Atacicept has the potential to block BLyS- and APRIL-mediated Ig isotope class switching and reduce IgG and IgA levels in patients with IgAN. This process may slow or prevent the formation and deposition of pathogenic Gd-IgA1 or immune complexes in the kidney and could ultimately improve outcomes for patients with IgAN. Agents directed at either BLyS or APRIL alone reduced immunoglobulin levels in healthy volunteers (VIS649) and patients with IgAN (BION-1301 and blisibimod).^27^, ^28^, ^29^

Here, we present the results of a phase II study of atacicept in patients with IgAN and persistent proteinuria, despite receiving optimal ACEi or ARB therapy. Key objectives were to evaluate the safety and tolerability, the pharmacodynamic effects on circulating Ig and Gd-IgA1, and the clinical response (proteinuria and renal function) of atacicept treatment for up to 72 weeks.

## Methods

### Study Design

JANUS was a randomized, double-blind, placebo-controlled phase II study of atacicept in patients with IgAN and persistent proteinuria (NCT02808429, Eudra CT 2016-002262-31). Eligible patients were randomized 1:1:1 to receive placebo, atacicept 25 mg, or atacicept 75 mg once weekly, administered by subcutaneous injections from prefilled 1 ml syringes. The planned treatment period was 72 weeks. Each patient entered a 24-week safety follow-up period following their last dose of treatment. See Supplementary Material for details on randomization and blinding.

An external independent data monitoring committee monitored patient safety regularly. After at least 5 patients per arm had completed 12 weeks’ treatment, cumulative safety data was assessed. An interim efficacy analysis was performed after 16 patients completed 24 weeks’ treatment. Following this analysis, the sponsor decided that owing to unexpectedly slow enrollment, the study would be terminated once all enrolled patients had completed at least 48 weeks’ treatment and the safety follow-up period. This decision was not related to the clinical safety or efficacy of atacicept.

All participants provided written informed consent before study initiation. Before commencement of the study at a given site, study protocol and consent forms were approved by the local independent ethics committee or institutional review board. The study was conducted in accordance with the principles of the Declaration of Helsinki and in compliance with the International Council for Harmonization E6 Guideline for Good Clinical Practice.

### Study Patients

Eligible patients were 18 years or older, with IgAN and persistent proteinuria despite being on a stable dose of ACEi and/or ARBs. Patients were required to initiate ACEi and/or ARB therapy at least 12 weeks before the screening visit, the dose of which must have been stable for at least 8 weeks before screening and considered optimal by the investigator. IgAN was confirmed by biopsy within the 60 months before screening, using the Oxford-MEST classification. UPCR ≥1 mg/mg by 24-hour urine collection during the screening period was required. Patients with UPCR ≥0.75 mg/mg at screening who had at least one documented UPCR ≥1 mg/mg within 12 months while on ACEi and/or ARB therapy were also eligible. The patients were required to have up-to-date vaccinations against pneumococcus and the influenza virus.

Patients with concomitant significant renal disease other than IgAN, including diabetic nephropathy, lupus nephritis, and Henoch Schönlein purpura, or with serum IgG levels <6 g/l were not eligible for the study. In addition, patients with >50% glomeruli with global glomerulosclerosis, or >50% cortical area involved by tubular atrophy or interstitial fibrosis were not eligible. Exclusion criteria also included those with estimated glomerular filtration rate (eGFR) of <25 ml/min per 1.73 m^2^ for patients with kidney biopsy performed within 3 months before screening, eGFR <35 ml/min per 1.73 m^2^ for patients with kidney biopsy performed between 3 and 12 months before screening, and eGFR <45 ml/min per 1.73 m^2^ for patients with kidney biopsy performed between 12 and 60 months before screening. Further exclusion criteria were blood pressure higher than 140/90 mmHg, an active infection or high infectious risk, prior cyclophosphamide or concomitant immunosuppressant use, and prior use of B-cell–directed biological therapies such as rituximab.

### Study Objectives and Assessments

The primary objective was to evaluate the safety and tolerability of atacicept in patients with IgAN and persistent proteinuria while on a stable dose of ACEi and/or ARB therapy. Safety was assessed based on the number of patients with TEAEs (MedDRA version 22.1), adverse events (AEs) leading to discontinuation, serious AEs, AEs leading to death, and AEs of special interest (AESIs). AESIs included cardiac failure, ischemic heart disease, cardiac arrhythmia, infections, hypersensitivity reactions (including anaphylactic/anaphylactoid shock conditions, asthma/bronchospasm, and angioedema), and injection-site reactions (captured when a patient experienced one or more of the following injection-site symptom: redness, bruising, swelling, induration, or itching). Physical assessments, clinically significant vital signs, electrocardiograms, and laboratory assessments were also evaluated.

Key secondary objectives were to evaluate the pharmacodynamic characteristics of atacicept, particularly the effect on circulating IgG, IgA, IgM, and Gd-IgA1 levels, as well as complement proteins C3 and C4. Blood samples were collected for analysis of these serum biomarkers at baseline and during treatment period.

Other objectives included assessment of clinical efficacy of atacicept compared with placebo on proteinuria, assessed by 24-hour urine collection at baseline and weeks 24, 48 and 72 of the treatment period, and renal function as assessed by eGFR at baseline and during treatment period. eGFR was calculated as per the Chronic Kidney Disease Epidemiology Collaboration for all patients other than those in Japan, where eGFR was calculated using the Japan Association of Chronic Kidney Disease formula 2008. Exploratory objectives included changes in the levels of immune cell subsets, including total B cells, mature naïve B cells, and memory B cells, which were evaluated by flow cytometry. See Supplementary Methods for details on statistical analyses.

## Results

### Study Population and Baseline Characteristics

**Figure 1:**
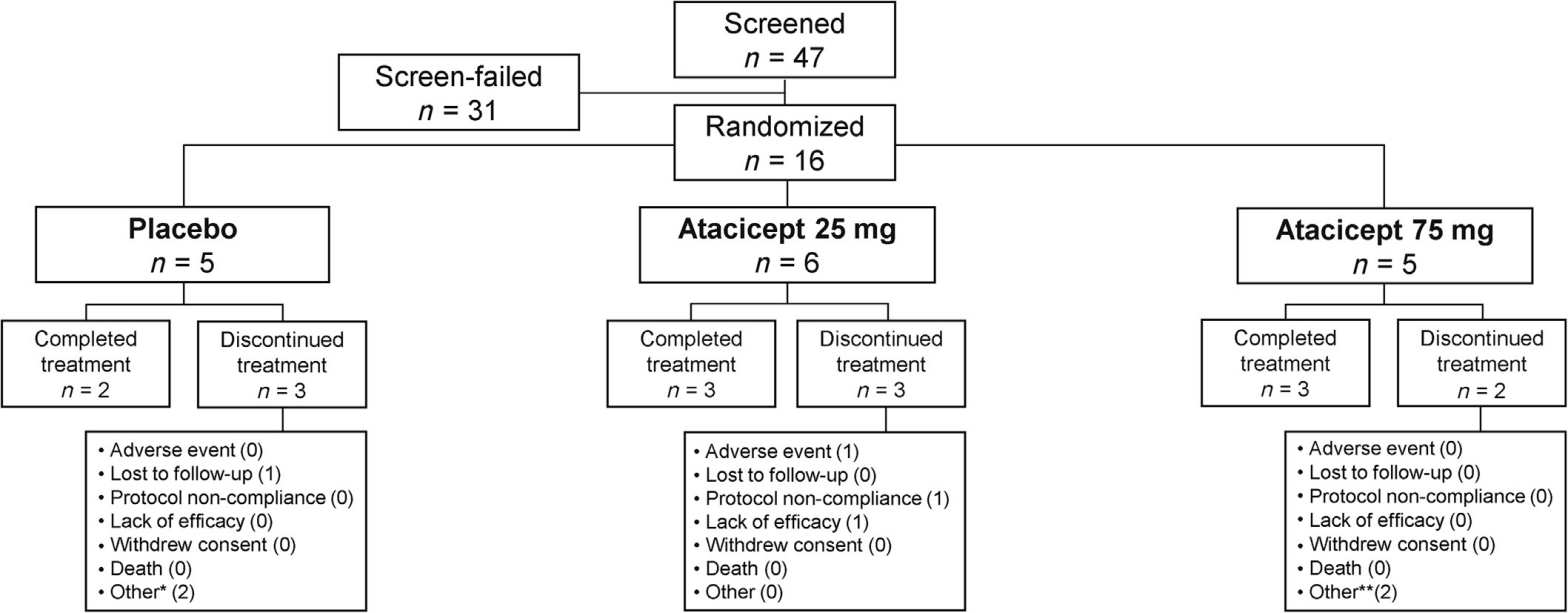
Patient disposition in the JANUS study. Of the 8 patients who discontinued treatment, 5 completed the safety follow-up period of 24 weeks (placebo, *n =* 2; atacicept 25 mg, *n =* 2; atacicept 75 mg, *n =* 1) and 1 patient completed a safety follow-up period of 12 weeks (placebo group). ∗Both due to study termination by sponsor. ∗∗One due to study termination by sponsor, one due to withdrawal from the study.

This study was conducted between January 2017 and February 2020. A total of 47 patients at 17 centers were screened for participation in the study, including 1 center in Japan, 1 in the United Kingdom, and 15 in the USA. Of these 47 patients, 16 were considered eligible to participate in the study and were randomized; 5 patients were randomized to receive placebo, 6 to atacicept 25 mg and 5 to atacicept 75 mg (Figure 1).

Most (69%) patients completed at least 60 weeks of study treatment. The median (quartile [Q]1, Q3) duration of treatment was 71.3 (67.1, 72.1) weeks for the placebo group, 52.6 (29.9, 73.1) weeks for the atacicept 25 mg, and 72.3 (63.1, 73.0) weeks for the atacicept 75 mg groups. The sponsor terminated the study early owing to slow enrollment, resulting in treatment discontinuation for 3 patients; 2 completed >60 weeks’ treatment and 1 completed >40 weeks’ treatment. All 3 patients completed 24 weeks of safety follow-up. A total of 5 patients discontinued treatment for other reasons (Figure 1).

The mean (SD) age of the study population was 43 (11.1) years and 50% were female (Table 1). The median (Q1, Q3) duration from diagnosis with IgAN was 2.38 (0.69, 4.2) years. All patients received concomitant ACEi and/or ARB treatment and had a recent kidney biopsy. The median time since most recent biopsy was 0.50 years in the placebo group, 1.80 years in the atacicept 25 mg group, and 0.97 years in the atacicept 75 mg group. Baseline median eGFR was similar across the groups (49–57 ml/min per 1.73 m^2^) and all patients had high proteinuria (median UPCR 1.4–1.8 mg/mg by 24-hour urine collection) despite maximal supportive therapy.

**Table 1:** Patient demographics and baseline characteristics (mITT population)

	Placebo (*n* = 5)	Atacicept 25 mg (*n* = 6)	Atacicept 75 mg (*n* = 5)
Age (years), mean ± SD	46 ± 3.1	41 ± 16.9	43 ± 8.9
Female, *n* (%)	1 (20)	5 (83)	2 (40)
Race, *n* (%)			
White	4 (80)	5 (83)	2 (40)
Black	0	0	0
Asian	1 (20)	1 (17)	1 (20)
Other	0	0	2 (40)
Ethnicity, Hispanic, *n* (%)	0	1 (17)	3 (60)
Time since diagnosis (years), median (Q1, Q3)	1.26 (1.05, 12.42)	2.17 (0.12, 2.99)	2.55 (2.52, 4.62)
Time since most recent kidney biopsy (years), median (Q1, Q3)	0.50 (0.31, 1.05)	1.80 (0.12, 2.96)	0.97 (0.33, 2.52)
History of tonsillectomy, *n* (%)	0	0	2 (40)
History of systemic corticosteroids, *n* (%)	1 (20)	2 (33)	1 (20)
Concomitant medications, *n* (%)			
ACEi and/or ARB	5 (100)	6 (100)	5 (100)
ACEi without ARB	3 (60)	3 (50)	1 (20)
ARB without ACEi	2 (40)	3 (50)	4 (80)
Diuretics	0	3 (50)	2 (40)
eGFR by CKD-EPI (ml/min per 1.73 m^2^), median (Q1, Q3)	49 (48, 54)	57 (53, 85)	55 (52, 92)
Proteinuria			
UPCR by 24-h urine collection (mg/mg), median (Q1, Q3)	1.6 (1.5, 1.6)	1.8 (0.8, 2.2)	1.4 (1.3, 1.7)
Total protein by 24-hr urine collection (g/d), median (Q1, Q3)	3.2 (2.3, 3.3)	2.1 (1.9, 2.9)	1.7 (1.6, 2.3)
Immunoglobulins			
IgA (g/l), mean ± SD	3.97 ± 1.72	3.58 ± 1.22	3.02 ± 0.85
IgG (g/l), mean ± SD	10.51 ± 2.63	9.45 ± 1.82	10.89 ± 1.10
IgM (g/l), mean ± SD	1.29 ± 0.51	0.90 ± 0.55	1.09 ± 0.30
Gd-IgA1 (ng/ml), mean ± SD	7690 ± 3642	6258 ± 3211	6052 ± 2773
Complement (mg/l)			
Serum C3, median (Q1, Q3)	1330 (1180, 1520)	1625 (1410, 1700)	1260 (1230, 1300)
Serum C4, median (Q1, Q3)	287 (282, 310)	332 (305,370)	379 (233, 408)

ACEi, angiotensin-converting-enzyme inhibitor; ARB, angiotensin receptor blocker; CKD-EPI, Chronic Kidney Disease Epidemiology Collaboration; eGFR, estimated glomerular filtration rate; Gd-IgA1, galactose-deficient IgA1; mITT, modified intent-to-treat; Q1/3, quartile 1/3; UPCR, urine protein:creatinine ratio.

### Safety

A total of 14 (88%) patients experienced ≥1 TEAEs throughout the study; 60% of patients in the atacicept 75 mg group and 100% of patients in both the placebo and atacicept 25 mg groups reported TEAEs (Table 2). All but 1 of TEAEs were mild or moderate in severity. One grade 3 event of cervical spinal stenosis was reported during the safety follow-up period by a patient who had received atacicept 25 mg.

**Table 2:** Summary of adverse events reported over the study period (mITT population)

Number (%) patients with	Placebo (*n =* 5)	Atacicept 25 mg (*n =* 6)	Atacicept 75 mg (*n =* 5)	Total (*N* = 16)
Any TEAEs	5 (100)	6 (100)	3 (60)	14 (88)
TEAEs during the treatment period	5 (100)	6 (100)	3 (60)	14 (88)
TEAEs in the post-treatment period	0	3 (50)	0	3 (19)
Mild TEAEs	5 (100)	6 (100)	3 (60)	14 (88)
Moderate TEAEs	2 (40)	5 (83)	1 (20)	8 (50)
Severe TEAEs	0	1 (17)	0	1 (6)
Treatment-related TEAEs	1 (20)	5 (83)	3 (60)	9 (56)
Serious TEAEs	1 (20)	3 (50)	0	4 (25)
Serious TEAEs in the treatment period	1 (20)	2 (33)	0	3 (19)
Serious TEAEs in the post-treatment period	0	1 (17)	0	1 (6)
TEAEs leading to treatment discontinuation	0	1 (17)	0	1 (6)
TEAEs with fatal outcome	0	0	0	0

mITT, modified intent-to-treat; TEAE, treatment-emergent adverse event.

TEAEs are defined as events occurring between the date of first dose and the end of the 24-week safety follow-up period. The treatment period is defined as the period between the date of first and last dose plus 7 days. Percentages are a proportion of the total number of patients in each column.

Administration-site conditions, including erythema (*n =* 5), pruritus (*n =* 3), bruising (n = 3) and injection-site reactions (*n =* 3), and infections/infestations, including urinary tract infection (*n =* 3), and viral gastroenteritis (*n =* 2), were the most reported TEAEs. Infections were reported in all groups (40% in placebo, 83% in atacicept 25 mg, and 20% in atacicept 75 mg groups).

A total of 9 patients reported TEAEs that were considered treatment related. The proportion of patients who reported treatment-related TEAEs was higher in the atacicept 25 mg (83%) and 75 mg (60%) groups than the placebo (20%) group. Most treatment-related TEAEs (54 of 56 events) were local conditions including injection-site erythema (atacicept 25 mg, *n =* 4; atacicept 75 mg, *n =* 1), injection-site reactions (atacicept 25 mg, *n =* 1; atacicept 75 mg, *n =* 2), injection-site pruritus (atacicept 25 mg, *n =* 3) and injection-site bruising (atacicept 25 mg, *n =* 2). Besides administration-site conditions, only 2 other treatment-related TEAEs were reported as follows: 1 case of herpes simplex in the placebo group and 1 case of pruritus in the atacicept 25 mg group.

Four patients reported at least 1 serious AE; 1 in the placebo group (ovarian adenoma) and 3 in the atacicept 25 mg group (1 patient with urinary bladder hemorrhage, 1 patient with 2 serious AEs: acute kidney injury and viral gastroenteritis, and 1 with cervical spinal stenosis), none of which were considered treatment related. All serious AEs occurred during the treatment period except cervical spinal stenosis that occurred >4 months after last treatment of atacicept 25 mg. One patient (17%) in the atacicept 25 mg group discontinued treatment due to a TEAE of injection-site pruritus. No deaths were reported.

All AESIs were mild or moderate. AESIs of injection-site reactions (≥1 injection-site symptom) were only reported by patients in the 2 atacicept groups, with higher incidence in the atacicept 25 mg group (*n =* 5, 83%) than the atacicept 75 mg group (*n =* 3, 60%). The proportion of patients who reported AESIs of infections was 40% (n = 2), 83% (*n =* 5), and 20% (*n =* 20) in placebo, atacicept 25 mg, and atacicept 75 mg groups, respectively. The incidence of hypersensitivity-related TEAEs was lower with placebo (*n =* 1, 20%) and atacicept 75 mg (*n =* 1, 20%) than with atacicept 25 mg (*n =* 4, 67%). Viral gastroenteritis was the only reported serious AESI of infection, reported by 1 patient who received atacicept 25 mg. No AESIs in the categories of cardiac failure, ischemic heart disease, cardiac arrhythmia, or demyelination were reported.

No cases of severe hypogammaglobulinemia (IgG <3 g/l) were reported. Serum IgG <6 g/l was observed in 1 patient in the atacicept 25 mg group and 2 patients in the atacicept 75 mg group. First, 1 patient receiving atacicept 25 mg had IgG <6 g/l at weeks 12 and 24 of the safety follow-up period; IgG levels were within the normal range during the treatment period. Second, 1 patient receiving atacicept 75 mg had IgG <6 g/l from weeks 8 to 72 of the treatment period; IgG levels increased to within the normal range by week 12 of the safety follow-up visit. A third patient had IgG <6 g/l at weeks 24 and 32. This patient withdrew from the study at week 33 and their serum IgG levels returned to the normal range by the early termination visit. No safety concerns emerged following a review of vital signs, electrocardiograms, or clinical laboratory assessments.

### Biomarkers

**Figure 2:**
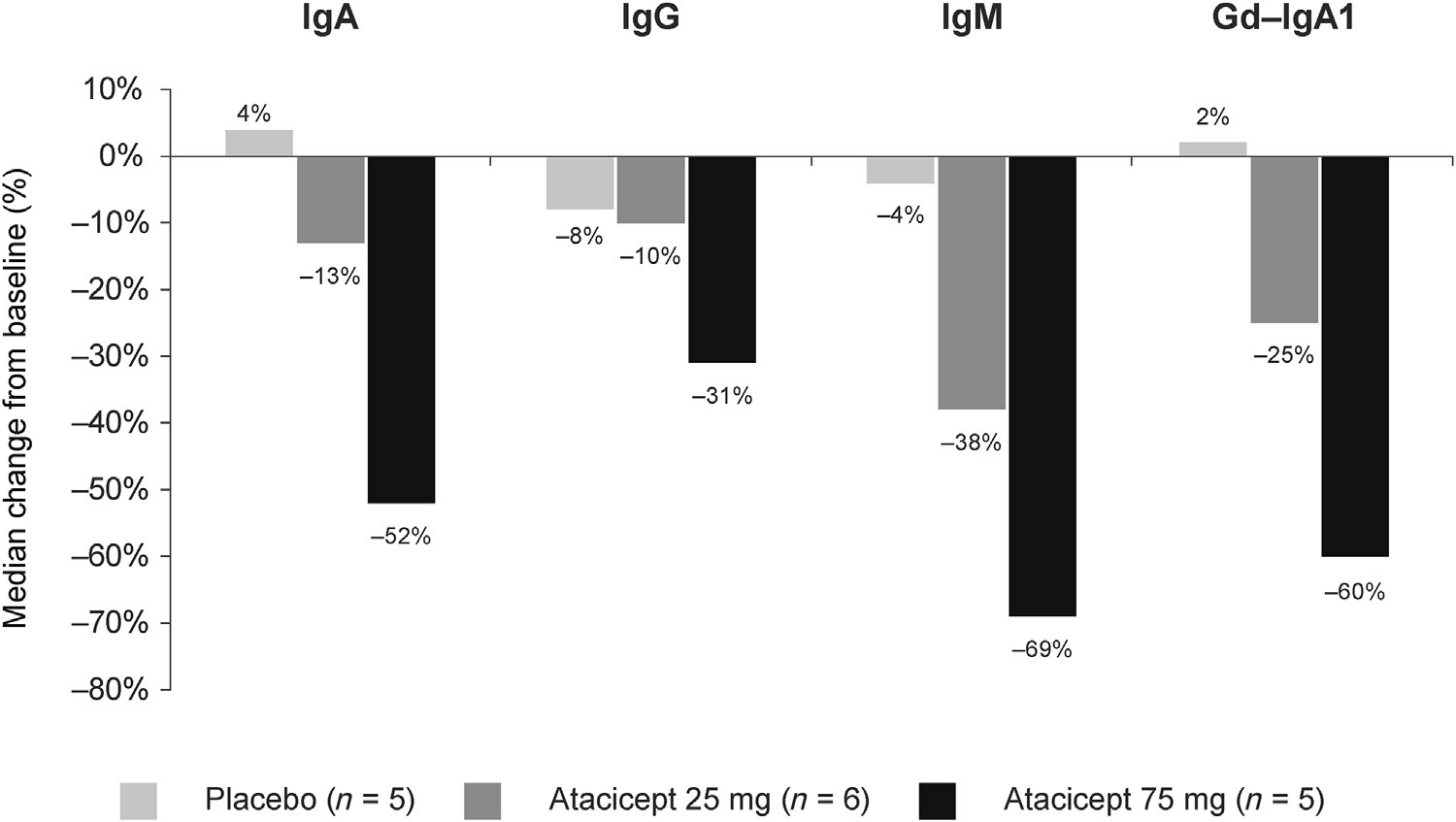
Median percentage change in serum IgA, IgG, IgM and Gd-IgA1 from baseline to week 24 (mITT population). Gd-IgA1, galactose-deficient IgA1; mITT, modified intent-to-treat

**Figure 3:**
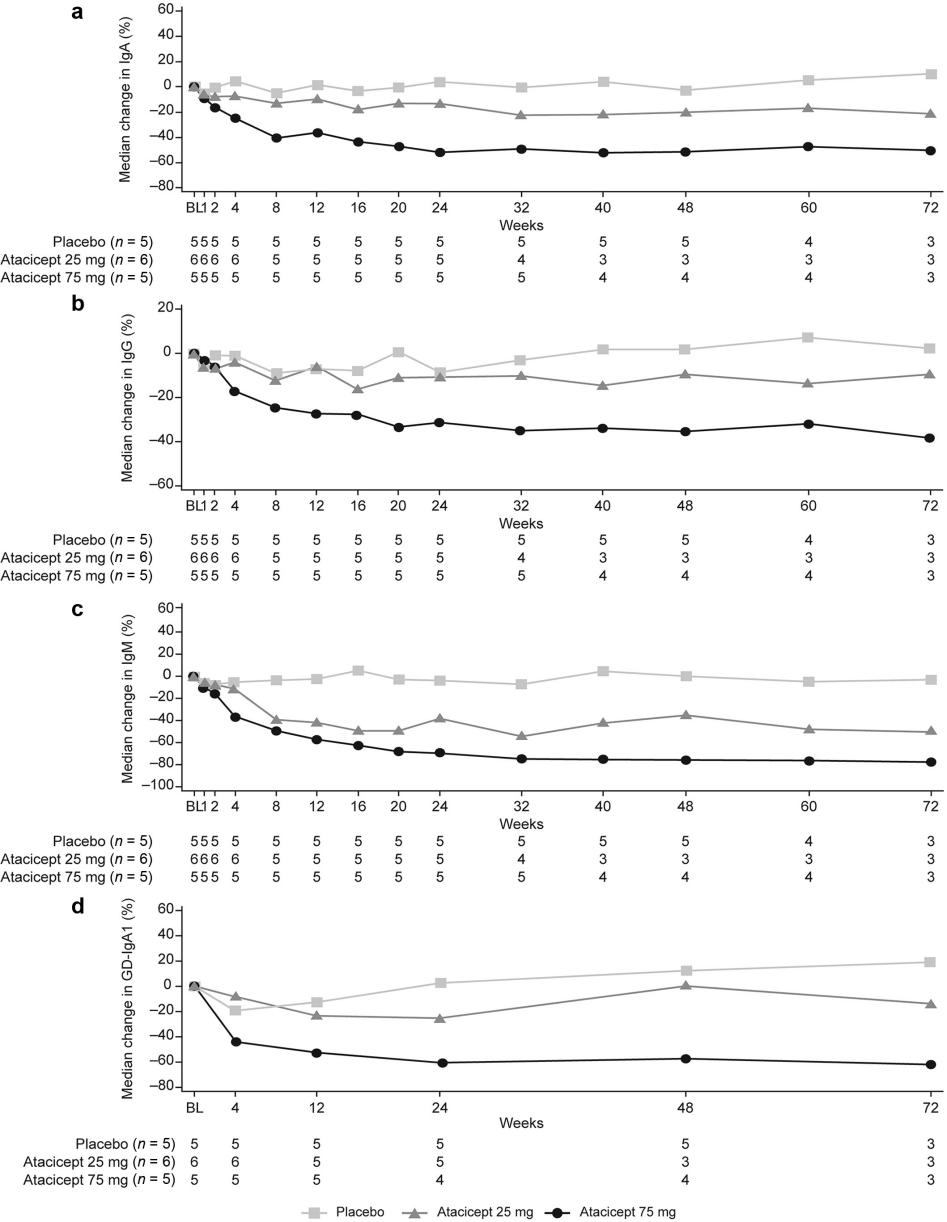
Median percentage change in (a) IgA, (b) IgG, (c) IgM, and (d) Gd-IgA1 from baseline to week 72 (mITT population). BL, baseline; Gd-IgA1, galactose-deficient IgA1; mITT, modified intent-to-treat.

A consistent, dose-dependent reduction in serum IgA, IgG and IgM was observed with atacicept from baseline to week 24 (Figure 2). The greatest reductions in IgA (placebo, −5%; atacicept 25 mg, −13%; atacicept 75 mg, −40%), IgG (placebo, −9%; atacicept 25 mg, −12%; atacicept 75 mg, −25%) and IgM (placebo −4%; atacicept 25 mg, −39%; atacicept 75 mg, −49%) occurred within the first 8 weeks of atacicept treatment. At week 24, median change from baseline in serum IgA levels was 4% with placebo, −13% with atacicept 25 mg, and −52% with atacicept 75 mg (Figure 2 and Supplementary Table S1). Corresponding changes with placebo, atacicept 25 mg, and atacicept 75 mg respectively in IgG were −8%, −10%, and −31%, and in IgM were −4%, −38%, and −69%. The serum levels of IgA, IgG, and IgM remained stable from weeks 24 to 72 (Figure 3 and Supplementary Table S1).

Similarly, there was a clear dose-dependent reduction in median serum Gd-IgA1 levels from baseline to week 24 with atacicept 25 mg (−25%) and atacicept 75 mg (−60%) compared with placebo (2%), which was generally maintained over time (Figures 2 and 3 and Supplementary Table S1). At week 72, median change from baseline in serum Gd-IgA1 levels were 19% with placebo, −14% with atacicept 25 mg and −61% with atacicept 75 mg.

Slight increases in serum C3 levels were observed with atacicept compared with placebo until week 24 and at week 48 (Supplementary Figure S1a). At week 72, C3 levels showed a decrease with atacicept 25 mg but continued to increase with atacicept 75 mg. Increases in serum C4 levels were observed in both atacicept groups compared with placebo, which showed a decrease in C4 levels until week 72 (Supplementary Figure S1b).

In the atacicept groups, a decrease in mature naïve B cells was observed until week 72 (Supplementary Table S2). No patterns of changes in the levels of other lymphocytes were observed across the study.

### Efficacy

**Figure 4:**
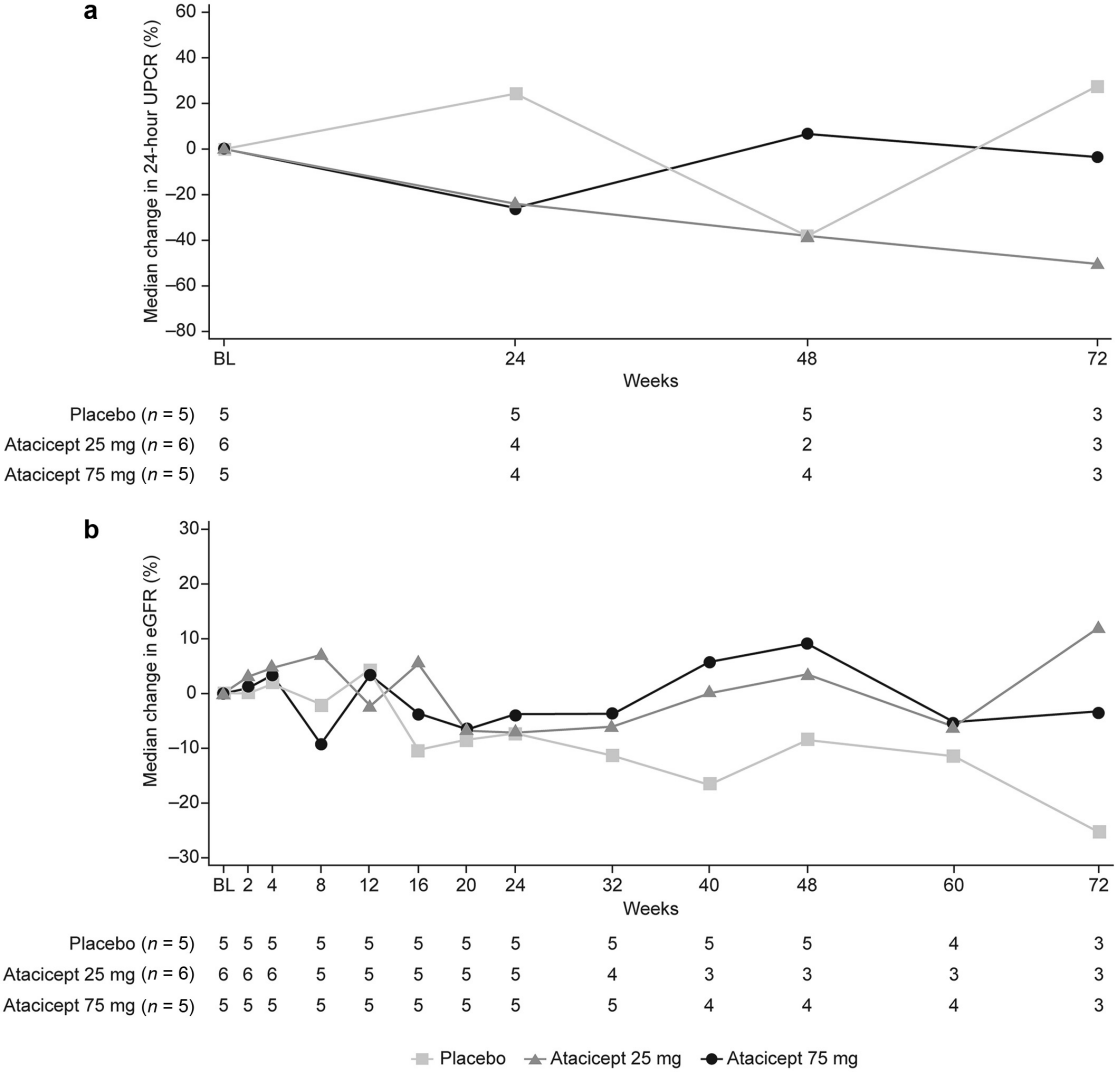
Median percentage change in (a) proteinuria (24-hour UPCR) and (b) eGFR from baseline to Week 72 (mITT population). BL, baseline; eGFR, estimated glomerular filtration rate; mITT, modified intent-to-treat; UPCR, urine protein-to-creatinine ratio.

At week 24, a reduction in proteinuria, as assessed by 24-hour UPCR, was observed for both atacicept groups; median change from baseline was −24% with atacicept 25 mg and −25% with atacicept 75 mg, compared with an increase of 24% with placebo (Supplementary Table S3). This persisted to week 48 (−38% from baseline) and week 72 (−50% from baseline) with atacicept 25 mg (Figure 4a). In the atacicept 75 mg group, the median change from baseline was 6.9% at week 48 and −3.2% at week 72. One patient in the atacicept 25 mg group and 2 patients in the atacicept 75 mg group with post-24-week data had increased proteinuria after week 24; 1 patient had worsening hypertension and type II diabetes, and the other 2 patients had increased proteinuria at week 48, which decreased at week 72. Further information is provided in the Supplementary Appendix.

There was a progressive decline in renal function assessed by eGFR in the placebo group; median change from baseline was −7% at week 24 (median [Q1, Q3] absolute change −4.0 [−10.0, −2.0] ml/min per 1.73 m^2^), −8% at week 48 (−3.0 [−11.0, −3.0] ml/min per 1.73 m^2^) and −25% at week 72 (−9.0 [−14.0, −2.0] ml/min per 1.73 m^2^; Figure 4b and Supplementary Table S3). Renal function was relatively stable with atacicept. Median change from baseline in eGFR with atacicept 25 mg was −7% at week 24, +3% at week 48, and +12% at week 72. Corresponding median (Q1, Q3) absolute change from baseline in eGFR with atacicept 25 mg was −4.0 (−6.0, −2.0) ml/min per 1.73 m^2^, 2.0 (−4.0, 3.0) ml/min per 1.73 m^2^, and 6.0 (6.0, 21.0) ml/min per 1.73 m^2^. Median change from baseline in eGFR with atacicept 75 mg was −4% at week 24, +9% at week 48 and −3% at week 72, with respective median (Q1, Q3) absolute changes of −2.0 (−17.0, 5.0) ml/min per 1.73 m^2^, 3.0 (−7.0, 15.5) ml/min per 1.73 m^2^, and −2.0 (−3.0, 9.0) ml/min per 1.73 m^2^.

*Post hoc* analysis of correlation between change from baseline in 24-hour UPCR and Gd-IgA1 at weeks 24, 48 and 72 was conducted (Supplementary Figure S2). This analysis suggested an association between reduced serum Gd-IgA1 levels and reduced proteinuria at the patient level with both atacicept doses.

## Discussion

There is a clinical need for targeted therapies that limit the progression of IgAN to ESRD. Atacicept is a novel immunomodulatory therapy that inhibits BLyS and APRIL signaling, which has been shown to reduce Ig levels in patients with autoimmune diseases, including systemic lupus erythematosus.^21^ Atacicept therefore, has the potential to halt the destructive inflammatory pathways that lead to the progressive damage of renal tissue in IgAN.

This phase II, randomized, double-blind, placebo-controlled study investigated the safety and efficacy of atacicept in patients with IgAN and persistent proteinuria. No new safety signals were identified and there were no deaths during the study. An acceptable safety profile with both atacicept doses was observed, with no dose effect on the frequency or severity of TEAEs. Administration-site reactions were observed with atacicept, but all were mild or moderate. Importantly, there was no increase in serious or severe TEAEs, including severe hypogammaglobulinemia, with long-term atacicept exposure. Although there was a limited number of patients in this study, the safety results were consistent with the established tolerability profile reported in an integrated pooled analysis of atacicept clinical trials including over 1500 patients.^30^

A dose-dependent decrease in serum pharmacodynamic biomarkers IgA, IgG, and IgM was observed with atacicept. The magnitude of IgA, IgG, and IgM reductions were similar to those observed in studies of atacicept in other indications. For example, in the ADDRESS II study of atacicept in patients with systemic lupus erythematosus, IgG changed by −25% with atacicept 75 mg at week 24 compared with −31% in this study. Results were comparable for IgA (−45% vs −52%) and IgM (−60% vs −69%).^21^

Importantly, atacicept treatment also led to dose-dependent reductions in the critical pathogenic IgAN serum biomarker Gd-IgA1, which changed by −25% and −60% at week 24 with atacicept 25 mg and 75 mg, respectively, and these reductions were generally maintained to week 72. To date, no other immunomodulatory agent has shown the same magnitude of effect on reducing serum Gd-IgA1 levels in patients with IgAN.

A clinically meaningful reduction in 24-hour proteinuria was observed at week 24 with atacicept, with continued improvement to week 72 with atacicept 25 mg. Despite a similar decrease in proteinuria at week 24 with atacicept 75 mg, this was not sustained over time. Proteinuria increased in 3 patients receiving atacicept at week 48, although these levels had reduced by week 72 in 2 of the patients. The proteinuria results in the atacicept 75 mg group are difficult to interpret given the small number of patients included and potential confounding factors (e.g., history of uncontrolled diabetes and hypertension). While there was a progressive decline in renal function by eGFR in the placebo group, both doses of atacicept resulted in stabilization of eGFR with improvements compared with placebo observed to week 72.

While the atacicept 25 mg group showed better improvement in proteinuria, the atacicept 75 mg group had greater reductions in serum Gd-IgA1, an autoantigen that plays a central role in the pathogenesis of IgAN. *Post hoc* analyses showed correlation between reduced Gd-IgA1 and improvement in proteinuria with both atacicept doses at the patient level. Due to the robust reduction in Gd-IgA1 with atacicept 75 mg, which is considered clinically meaningful by the authors, and the overall trend for improvements in eGFR, these results provide important corroborative evidence for the potential benefit of atacicept for IgAN patients. Further investigation of additional atacicept doses that have a greater effect on pharmacodynamic parameters is warranted.

The main limitation of this study was the small sample size; the results should therefore be interpreted with caution. The independent data monitoring committee provided recommendation to open enrollment for an atacicept 150 mg group, but this was not initiated due to the early termination of the trial. The early termination precluded longer-term assessments of the efficacy and safety of atacicept in patients with IgAN. A larger study of longer duration with inclusion of atacicept 150 mg is being conducted to confirm the findings reported here (the ORIGIN phase IIb study, NCT04716231).

In conclusion, in the phase II JANUS trial, atacicept demonstrated an acceptable safety profile in patients with IgAN and persistent proteinuria, with meaningful changes in 24-hour proteinuria, potential stabilization of renal function, and substantial reductions in the levels of Gd-IgA1 compared with placebo. Although undertaken in a small number of patients, these results suggest that the benefit–risk profile of atacicept is acceptable for patients with IgAN who are at risk of renal failure, providing early proof of concept that warrants further clinical investigation.

## Appendix

### JANUS Study Investigators

Radica Alicic (Providence Sacred Heart Medical Center & Children’s Hospital, USA), Gerald Appel (Columbia University Medical Center, USA), Syed Babar (Southeastern Clinical Research Institute, LLC, USA), Jonathon Barratt (University Hospitals of Leicester NHS Trust, UK), Diogo Belo (California Institute of Renal Research, USA), Yongen Chang (University of California Irvine Health, USA), Scott Cohen (Medical Faculty Associates, Inc., USA), Claude Galphin (Southeast Renal Research Institute, USA), Gregory Greenwood (Brookview Hills Research Associates, LLC, USA), Nelson Kopyt (Northeast Clinical Research Center, LLC, USA), Sumit Kumar (Nephrotex Research Group, USA), Bradley Marder (Colorado Kidney Care PC, USA), Brad Rovin (Ohio State University Wexner Medical Center, USA), Peter Santos (AKDHC Medical Research Services, LLC, USA), Meghan Sise (Massachusetts General Hospital Renal Clinic, USA), Yusuke Suzuki (Juntendo University Hospital, Japan), James Tumlin (GA Nephrology Research Institute, USA)

## Disclosure

JB is a consultant and Advisory Board/Study Steering Committee member for EMD Serono. JT is a consultant to and received grant funding from EMD Serono. YS is a consultant to Merck KGaA, Darmstadt, Germany, EMD Serono, Kyowa Kirin Ltd., and Novartis Pharmaceuticals, received research funding from EMD Serono, and has conducted collaborative studies with Visterra Inc., Moderna Inc., Retrophin Inc. and, Chinook Therapeutics. AK, AA and KP are employed by EMD Serono. HJ is employed by the healthcare business of Merck KGaA, Darmstadt, Germany; and HG is employed by Merck KGaA, Darmstadt, Germany. GA is a consultant to EMD Serono and conducted research studies with EMD Serono, Calliditas, and Retrophin Inc.

## Supplementary Material

Supplementary File (PDF)

**Supplementary Methods.** Randomization and blinding, Statistical methods.

**Table S1.** Median change from baseline in serum Gd-IgA1, IgA, IgG and IgM at Weeks 24, 48 and 72 (mITT population).

**Table S2.** Median change from baseline in total B cells, memory B cells and mature naive B cells at Weeks 24, 48 and 72 (FC population).

**Table S3.** Median change from baseline in 24-hour UPCR, total protein and eGFR at Weeks 24, 48 and 72 (mITT population).

**Figure S1.** Median percentage change in serum (A) C3 and (B) C4 levels from baseline to Week 72 (mITT population).

**Figure S2.** Post-hoc analysis of correlation between percentage change from baseline of 24-hour UPCR and Gd-IgA1 at (A) Week 24, (B) Week 48 and (C) Week 72 (mITT population).

**Supplementary Appendix**. Patient narratives for those with increased proteinuria with atacicept.

**CONSORT checklist.**
