# Supplementary material for: Randomized Phase II JANUS Study of Atacicept in Patients With IgA Nephropathy and Persistent Proteinuria
Source: Kidney Int Rep. 2022 May 26;7(8):1831–41. doi: 10.1016/j.ekir.2022.05.017 (PMC9366370; doi:10.1016/j.ekir.2022.05.017)
Supplement: Supplementary File (PDF) [file mmc1.pdf]

SUPPLEMENTARY MATERIALS

Supplementary Methods

Randomization and blinding

Patients were recruited by study investigators and randomized through a central randomization process by an Interactive Web Response System, delegated by the sponsor to Cenduit Solutions, North Carolina, US. Randomization was stratified according to baseline proteinuria (UPCR <2 mg/mg vs ≥2 mg/mg at screening) and race (Asian vs non-Asian) and conducted in permuted blocks. The first two doses of study treatment were administered at the study site. At the Week 2 visit, the treatment was dispensed to allow for self-administration from Week 3 onwards. All data were collected at scheduled visits to the study sites.

The double-blind approach was strictly maintained for all patients and study sites during the treatment and safety follow-up periods. Sponsor and contract-research organization staff involved in the conduct of the study remained blinded to individual patient data. The interim analysis was performed by an Unblinded Firewall Team who were not further involved in the conduct or analysis of the study in order to preserve the integrity of the primary analysis. After Day 1, results of analyses that could reveal the PD effects of atacicept in an individual patient (e.g. serum Ig levels) were blinded to the study site, sponsor and contract-research organization.

Statistical methods

All randomized patients received at least one dose of study medication (modified intent-to-treat [mITT] population) and had at least one post-dose assessment (safety population),

therefore the safety and mITT populations are identical. The flow cytometry (FC) population included all patients in the safety population who were part of a site selected for FC analysis and had at least one evaluable FC sample. All results are reported as mITT, other than FC parameters which are reported for the FC population.

Based on observations in the study of atacicept in patients with SLE (ADDRESS II), a sample size of 10 patients per arm for the final analysis was deemed sufficient to capture a treatment effect in terms of safety and PD; this was not based on statistical power since no hypotheses were tested. An interim analysis of proteinuria, biomarkers and safety was performed after 16 patients had completed 24 weeks of treatment, with the final analysis performed when all patients had completed at least 48 weeks of treatment plus 24 weeks of safety follow up. All data are summarized using descriptive statistics.

Supplementary Tables and Figures

Supplementary Table S1. Median change from baseline in serum Gd-IgA1, IgA, IgG and IgM at Weeks 24, 48 and 72 (mITT population)

	**Placebo (N = 5)**	**Atacicept 25 mg (N = 6)**	**Atacicept 75 mg (N = 5)**
**Gd-IgA1**, median (Q1, Q3)			
**Absolute levels at baseline (ng/mL; n = 5, 6, 5)**	8100 (4330, 9500)	5715 (3750, 9010)	5250 (4350, 8750)
**Week 24** (n = 5, 5, 4)			
Absolute levels (ng/mL)	9590 (4130, 9730)	5770 (3830, 5800)	3085 (1976, 3660)
Percentage change from baseline	2 (-3, 5)	-25 (-26, -24)	-60 (-66, -47)
**Week 48** (n = 5, 3, 4)			
Absolute levels (ng/mL)	8540 (6570, 8750)	7670 (3740, 7740)	2570 (1590, 3110)
Percentage change from baseline	12 (-10, 52)	0 (-15, 1)	-57 (-63, -47)
**Week 72** (n = 3, 3, 3)			
Absolute levels (ng/mL)	10200 (6670, 11300)	5120 (3570, 7750)	1700 (843, 3750)
Percentage change from baseline	19 (-19, 54)	-14 (-33, -5)	-61 (-70, -57)
**IgA**, median (Q1, Q3)			
**Absolute levels at baseline (ng/mL; n = 5, 6, 5)**	3.8 (3.1, 5.1)	3.9 (2.3, 4.2)	3.3 (2.3, 3.3)
**Week 24** (n = 5, 5, 5)			
Absolute levels (g/L)	3.9 (3.2, 5.0)	3.7 (3.1, 3.9)	1.6 (1.3, 2.0)
Percentage change from baseline	3.9 (3.2, 10.9)	-13.3 (-15.7, -8.3)	-51.9 (-53.0, 41.2)

**Week 48** (n = 5, 3, 4)			
Absolute levels (g/L)	3.7 (2.9, 4.8)	3.9 (2.9, 4.1)	1.4 (1.1, 1.6)
Percentage change from baseline	-2.9 (-6.2, 30.1)	-20.2 (-20.7, -7.6)	-51.7 (-58.0, -41.8)
**Week 72** (n = 3, 3, 3)			
Absolute levels (g/L)	3.4 (2.4, 5.1)	3.2 (2.9, 4.1)	1.5 (0.8, 1.6)
Percentage change from baseline	10.2 (-1.2, 38.1)	-21.3 (-24.9, -19.6)	-50.3 (-62.4, -33.63)
**IgG**, median (Q1, Q3)			
**Absolute levels at baseline (ng/mL; n = 5, 6, 5)**	9.5 (8.8, 11.6)	9.3 (7.7, 10.1)	10.8 (10.0, 11.1)
**Week 24** (n = 5, 5, 5)			
Absolute levels (g/L)	10.1 (8.1, 10.6)	8.8 (7.1, 9.0)	7.4 (5.8, 8.4)
Percentage change from baseline	-8.2 (-8.9, -5.3)	-9.7 (-10.4, -8.7)	-31.3 (-46.4, -25.8)
**Week 48** (n = 5, 3, 4)			
Absolute levels (g/L)	11.5 (8.9, 12.2)	8.8 (7.9, 11.4)	7.1 (6.0, 8.4)
Percentage change from baseline	1.8 (-1.0, 2.2)	-9.3 (-9.3, 3.5)	-35.3 (-39.9, -29.5)
**Week 72** (n = 3, 3, 3)			
Absolute levels (g/L)	9.0 (9.0, 12.9)	8.3 (7.7, 11.4)	6.2 (5.8, 8.6)
Percentage change from baseline	2.3 (-11.8, 11.3)	-9.5 (-14.8, 0.3)	-38.1 (-41.6, -22.5)

**IgM**, median (Q1, Q3)			
**Absolute levels at baseline (ng/mL; n = 5, 6, 5)**	1.0 (0.9, 1.7)	0.8 (0.4, 1.4)	1.0 (1.0, 1.3)
**Week 24** (n = 5, 5, 5)			
Absolute levels (g/L)	1.1 (1.0, 1.5)	0.4 (0.3, 0.5)	0.3 (0.3, 0.3)
Percentage change from baseline	-4.0 (-9.2, 4.5)	-38.1 (-46.5, -37.3)	-69.2 (-74.5, -66.0)
**Week 48** (n = 5, 3, 4)			
Absolute levels (g/L)	1.0 (0.9, 1.5)	0.2 (0.2, 0.6)	0.2 (0.2, 0.3)
Percentage change from baseline	0.0 (-9.6, 5.4)	-35.4 (-54.8, -30.3)	-75.9 (-79.2, -72.6)
**Week 72** (n = 3, 3, 3)			
Absolute levels (g/L)	1.0 (0.8, 1.7)	0.2 (0.2, 0.3)	0.2 (0.2, 0.4)
Percentage change from baseline	-3.4 (-4.0, -0.6)	-50.0 (-67.7, -39.4)	-77.5 (-84.3, -71.3)

Gd-IgA1, galactose-deficient immunoglobulin A1; Ig, immunoglobulin; mITT, modified intent-to-treat; Q1/3, quartile 1/3

Supplementary Table S2. Median change from baseline in total B cells, memory B cells and mature naive B cells at Weeks 24, 48 and 72 (FC population)

	**Placebo (N = 3)**	**Atacicept 25 mg (N = 3)**	**Atacicept 75 mg (N = 3)**
**Total B cells (assay with CD45)**, median (Q1, Q3)			
**Absolute levels at baseline (n = 2, 3, 3)**	9.2 (5.2, 13.2)	10.9 (7.8, 11.4)	6.3 (5.3, 19.0)
**Week 24** (n = 2, 2, 2)			
Absolute levels	8.5 (4.6, 12.3)	6.4 (6.0, 6.7)	9.9 (7.2, 12.5)
Percentage change from baseline	-9.2 (-11.5, -6.8)	-30.7(-47.4, -14.1)	0.8 (-34.2, 35.9)
**Week 48** (n = 2, 1, 2)			
Absolute levels	9.4 (5.3, 13.4)	2.9 (2.9, 2.9)	10.3 (7.4, 13.2)
Percentage change from baseline	1.7 (1.5, 1.9)	-62.8 (-62.8, -62.8)	-6.5 (-30.5, 17.5)
**Week 72** (n = 1, 0, 2)			
Absolute levels	4.7 (4.7, 4.7)	–	8.8 (5.8, 11.8)
Percentage change from baseline	-9.6 (-9.6, -9.6)	–	-22.9 (-37.9, -7.9)
**Mature naive B cells**, median (Q1, Q3)			
**Absolute levels at baseline (n = 2, 3, 3)**	7.7 (3.5, 11.8)	8.9 (5.2, 9.3)	4.7 (3.2, 10.9)
**Week 24** (n = 2, 2, 2)			
Absolute levels	6.9 (3.0, 10.8)	2.8 (1.1, 4.4)	2.6 (2.2, 2.9)
Percentage change from baseline	-11.4 (-14.3, -8.5)	-65.8 (-78.9, -52.7)	-52.3 (-73.4, -31.3)
**Week 48** (n = 2, 1, 2)			

Absolute levels	7.7 (3.5, 11.9)	0.4 (0.4, 0.4)	3.1 (2.9, 3.3)
Percentage change from baseline	0.42 (0.0, 0.9)	-92.3 (-92.3, -92.3)	-51.6 (-73.4, -29.8)
**Week 72** (n = 1, 0, 2)			
Absolute levels	3.0 (3.0, 3.0)	–	2.7 (2.4, 2.9)
Percentage change from baseline	-14.3 (-14.3, -14.3)	–	-58.1 (-78.0, -38.3)
**Memory B cells**, median (Q1, Q3)			
**Absolute levels at baseline (n = 2, 2, 3)**	0.6 (0.4, 0.7)	0.5 (0.5, 0.5)	0.9 (0.6, 3.6)
**Week 24** (n = 2, 2, 2)			
Absolute levels	0.5 (0.2, 0.7)	0.8 (0.6, 0.9)	2.4 (1.9, 2.9)
Percentage change from baseline	-25.0 (-50.0, 0.0)	80.0 (80.0, 80.0)	45.8 (-19.4, 111.1)
**Week 48** (n = 2, 1, 2)			
Absolute levels	0.5 (0.4, 0.6)	0.4 (0.4, 0.4)	2.2 (1.2, 3.1)
Percentage change from baseline	-7.1 (-14.3, 0.0)	-20.0 (-20.0, -20.0)	43.1 (-13.9, 100.0)
**Week 72** (n = 1, 0, 2)			
Absolute levels	0.2 (0.2, 0.2)	–	1.9 (0.9, 2.8)
Percentage change from baseline	-50.0 (-50.0, -50.0)	–	13.9 (-22.2, 50.0)

FC, flow cytometry; Q1/3, quartile 1/3

Supplementary Table S3. Median change from baseline in 24-hour UPCR, total protein and eGFR at Weeks 24, 48 and 72 (mITT population)

**Median (Q1, Q3)**	**Placebo (N = 5)**	**Atacicept 25 mg (N = 6)**	**Atacicept 75 mg (N = 5)**
**UPCR by 24-hour urine collection**			
**Absolute levels at baseline** (mg/mg; n = 5, 6, 5)	1.6 (1.5, 1.6)	1.8 (0.8, 2.2)	1.4 (1.3, 1.7)
**Week 24** (n = 5, 4, 4)			
Absolute levels(mg/mg)	2.0 (1.2, 2.7)	2.2 (1.0, 2.9)	0.9 (0.7, 1.6)
Percentage change from baseline	24.5 (-24.3, 64.2)	-23.6 (-60.5, 16.9)	-25.3 (-40.8, -14.5)
**Week 48** (n = 5, 2, 4)			
Absolute levels (mg/mg)	1.1 (1.0, 2.2)	2.0 (1.2, 2.7)	1.3 (1.0, 2.3)
Percentage change from baseline	-37.6 (-44.6, 37.7)	-37.6 (-44.1, -31.0)	6.9 (-35.5, 40.0)
**Week 72** (n = 3, 3, 3)			
Absolute levels (mg/mg)	2.5 (0.9, 2.8)	1.1 (0.2, 3.6)	1.6 (1.2, 2.2)
Percentage change from baseline	27.8 (-40.7, 73.5)	-50.1 (-73.6, -8.2)	-3.2 (-30.2, 73.3)
**Total protein by 24-hour urine collection**			
**Absolute levels at baseline** (g/day; n = 5, 6, 5)	3.2 (2.3, 3.3)	2.1 (1.9, 2.9)	1.7 (1.6, 2.3)
**Week 24** (n = 5, 4, 4)			
Absolute levels (g/day)	4.5 (3.4, 5.0)	3.5 (1.8, 3.7)	1.5 (0.6, 3.2)
Percentage change from baseline	14.9 (3.7, 61.9)	-8.5 (-63.4, 36.7)	-29.0 (-49.4, 19.1)
**Week 48** (n = 5, 2, 4)			
Absolute levels (g/day)	2.4 (1.5, 4.5)	2.4 (1.2, 3.7)	2.1 (0.9, 4.0)

Percentage change from baseline	-27.7 (-51.2, 13.5)	-44.2 (-49.8, -38.6)	27.0 (-30.7, 75.2)
**Week 72** (n = 3, 3, 3)			
Absolute levels (g/day)	2.9 (2.7, 6.0)	1.0 (0.2, 5.9)	2.6 (1.8, 2.8)
Percentage change from baseline	-8.7 (-19.0, 53.1)	-57.2 (-84.3, -1.7)	10.7 (-2.4, 56.2)
**eGFR by CKD-EPI**			
**Absolute levels at baseline** (mL/min/1.73m^2; n = 5, 6, 5)	49.0 (48.0, 54.0)	57.0 (53.0, 85.0)	55.0 (52.0, 92.0)
**Week 24** (n = 5, 5, 5)			
Absolute levels (mL/min/1.73m^2)	40.0 (38.0, 47.0)	52.0 (47.0, 60.0)	50.0 (38.0, 74.0)
Percentage change from baseline	-7.4 (-20.8, -4.1)	-7.1 (-11.3, -2.4)	-3.8 (-20.4, 13.0)
**Week 48** (n = 5, 3, 4)			
Absolute levels (mL/min/1.73m^2)	39.0 (37.0, 46.0)	56.0 (47.0, 60.0)	65.5 (43.5, 98.0)
Percentage change from baseline	-8.3 (-22.9, -6.1)	3.4 (-7.8, 5.7)	9.1 (-8.8, 27.5)
**Week 72** (n = 3, 3, 3)			
Absolute levels (mL/min/1.73m^2)	34.0 (27.0, 52.0)	59.0 (57.0, 79.0)	50.0 (38.0, 89.0)
Percentage change from baseline	-25.0 (-29.2, -3.7)	11.8 (11.3, 36.2)	-3.3 (-3.8, 31.0)

CKD-EPI, Chronic Kidney Disease Epidemiology Collaboration; eGFR, estimated glomerular filtration rate; mITT, modified intent-to-treat; Q1/3, quartile 1/3; UPCR, urine protein:creatinine ratio

Supplementary Figure S1. Median percentage change in serum (A) C3 and (B) C4 levels from baseline to Week 72 (mITT population)

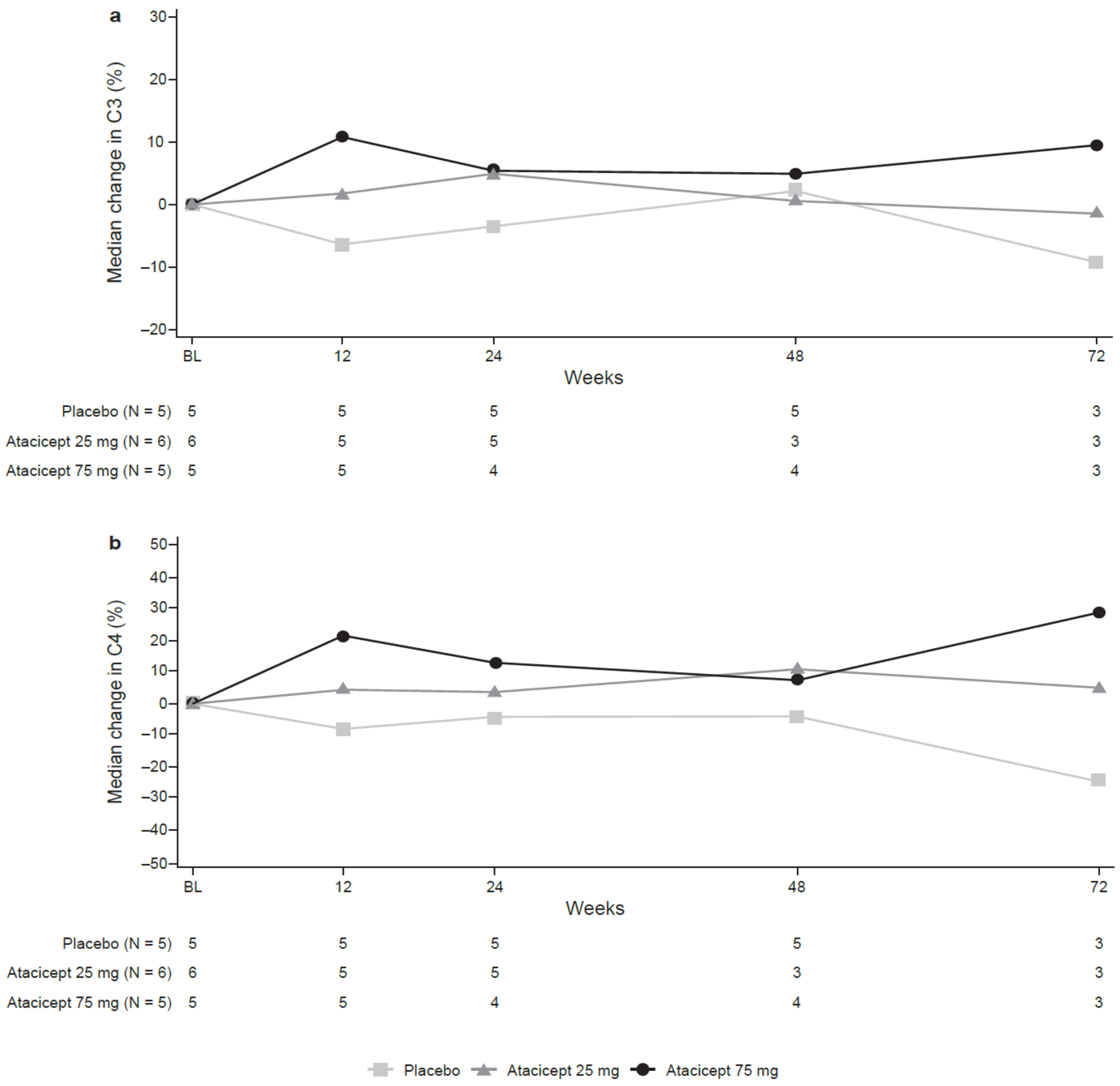

C3, complement component 3; C4, complement component 4; mITT, modified intent-to-treat

Supplementary Figure S2. Post-hoc analysis of correlation between percentage change from baseline of 24-hour UPCR and Gd-IgA1 at (A) Week 24, (B) Week 48 and (C) Week 72 (mITT population)

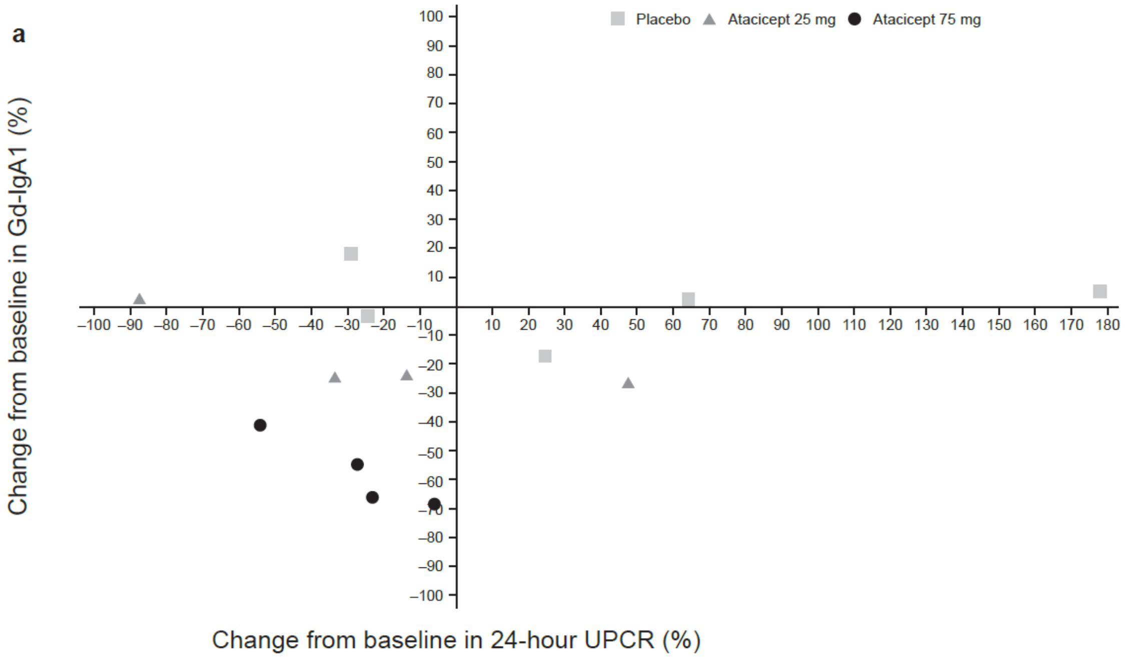

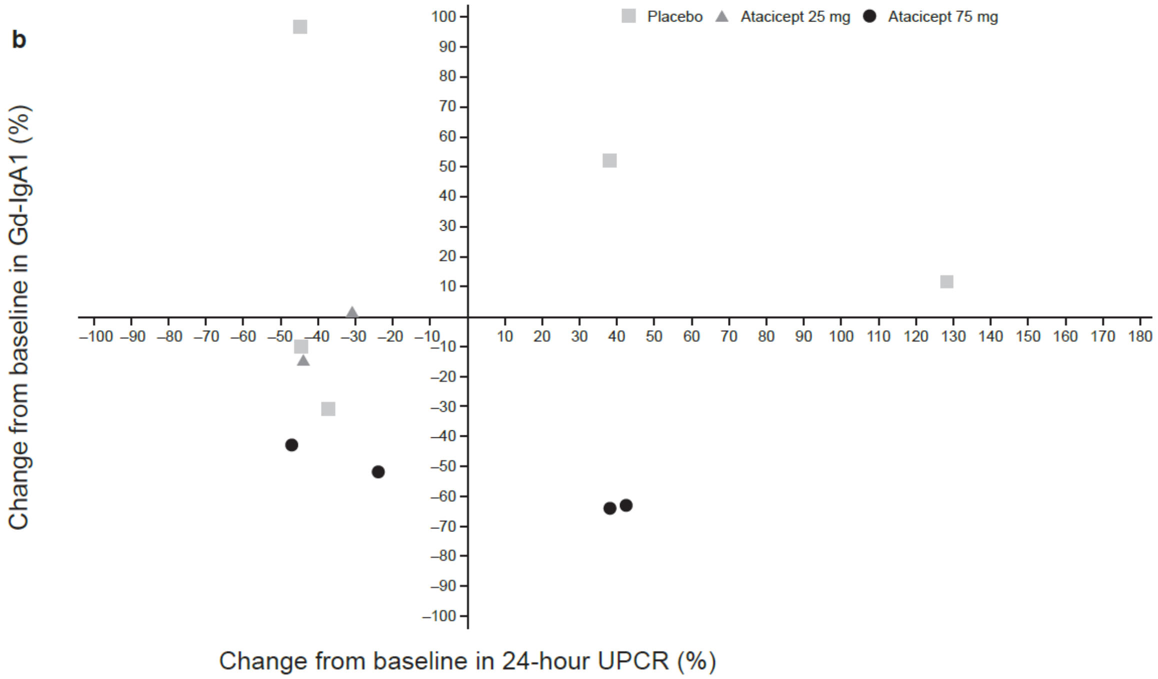

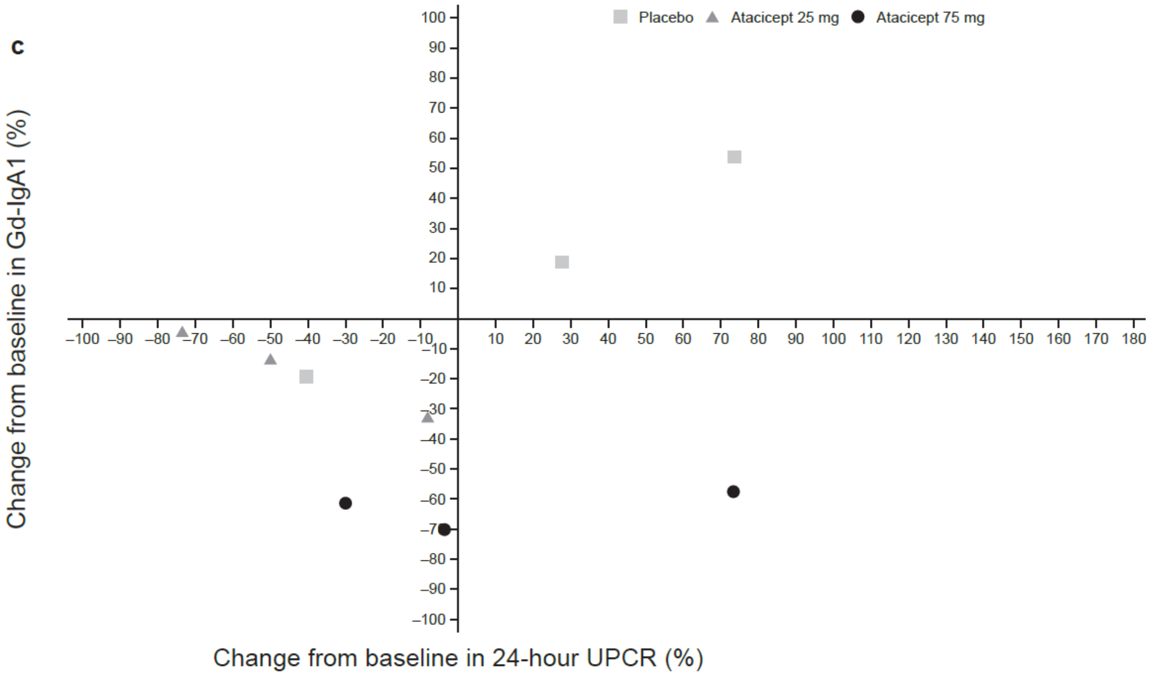

GD-IgA1, galactose-deficient immunoglobulin A1; UPCR, urine protein:creatinine ratio; mITT, modified intent-to-treat

Supplementary Appendix

Patient narratives for those with increased proteinuria with atacicept

One patient in the atacicept 25 mg group and two patients in the atacicept 75 mg group experienced increased proteinuria during the treatment period of the study.

Firstly, a patient in the atacicept 25 mg group had previously received treatment for anemia, a disease-related condition. Improvements in proteinuria, as measured by 24-hour UPCR, were observed with atacicept 25 mg at Week 24. At Week 48, 24-hour UPCR increased but reduced at a Week 48 unscheduled visit. At the end of the treatment period, 24-hour UPCR was higher than the Week 48 unscheduled visit, but lower than baseline of the study.

Change from baseline in 24-hour UPCR (mg/mg):

Study visit	Date of collection (relative day)	Analysis result	Change from baseline
Screening	-15	3.683	–
Week 0/Day 1	1	4.108	–
Week 24	169	2.592	-1.303
Week 48	340	4.209	0.313
Week 48*	340	2.687	-1.209
Week 72	508	3.576	-0.319

*Unscheduled visit

Around the time of increased proteinuria, the patient experienced a mild case of anemia that was considered unrelated to the study medication and did not require treatment.

Secondly, a patient in the atacicept 75 mg group had a history of hypertension, which was considered moderate in severity and related to the study condition, as well as type II diabetes. Improvement in this patient’s proteinuria was observed at Week 24. However, 24-hour UPCR increased at Week 48 and at Week 72.

Change from baseline in 24-hour UPCR (mg/mg):

Study visit	Date of collection (relative day)	Analysis result	Change from baseline
Screening	-15	1.068	–
Week 0/Day 1	1	0.814	–
Week 24	164	0.722	-0.219
Week 48	336	1.297	0.356
Week 72	504	1.631	0.690

The patient required insulin shortly after the increase in proteinuria due to an adverse event of uncontrolled type II diabetes mellitus. Treatment was ongoing at the end of the study. The patient also required treatment for exacerbated hypertension twice during the treatment period.

Finally, a patient who received atacicept 75 mg had increased 24-hour UPCR at Week 48, which reduced by Week 72 to similar levels as those at the start of the study. This patient did not report any adverse events during the study.

Change from baseline in 24-hour UPCR (mg/mg):

Study visit	Date of collection (relative day)	Analysis result	Change from baseline
Screening	-21	2.350	–
Week 0	-1	2.238	–
Week 24	168	2.161	-0.133
Week 48	336	3.260	0.966
Week 72	503	2.220	-0.074

CONSORT 2010 checklist of information to include when reporting a randomised trial*

Section/Topic	Item No	Checklist item	Reported on page No
**Title and abstract**			
	1a	Identification as a randomised trial in the title	1 (line 3)
	1b	Structured summary of trial design, methods, results, and conclusions (for specific guidance see CONSORT for abstracts)	2 (unstructured per journal requirements)
**Introduction**			
Background and objectives	2a	Scientific background and explanation of rationale	4-5 (line 78-97)
	2b	Specific objectives or hypotheses	5 (line 98-102)
**Methods**			
Trial design	3a	Description of trial design (such as parallel, factorial) including allocation ratio	5 (line 105-111)
	3b	Important changes to methods after trial commencement (such as eligibility criteria), with reasons	5-6 (line 112-118)
Participants	4a	Eligibility criteria for participants	6 (line 126-135)

	4b	Settings and locations where the data were collected	26 (line 533-534)
Interventions	5	The interventions for each group with sufficient details to allow replication, including how and when they were actually administered	5 (line 107-108)<br>26 (line 531-534)
Outcomes	6a	Completely defined pre-specified primary and secondary outcome measures, including how and when they were assessed	7-8 (line 150-164)
	6b	Any changes to trial outcomes after the trial commenced, with reasons	N/A
Sample size	7a	How sample size was determined	27 (line 551-554)
	7b	When applicable, explanation of any interim analyses and stopping guidelines	27 (line 554-557)
Randomisation:			
Sequence generation	8a	Method used to generate the random allocation sequence	26 (line 527-531)

	8b	Type of randomisation; details of any restriction (such as blocking and block size)	26 (line 527-531)
Allocation concealment mechanism	9	Mechanism used to implement the random allocation sequence (such as sequentially numbered containers), describing any steps taken to conceal the sequence until interventions were assigned	26 (line 527-531)
Implementation	10	Who generated the random allocation sequence, who enrolled participants, and who assigned participants to interventions	26 (line 527-531)
Blinding	11a	If done, who was blinded after assignment to interventions (for example, participants, care providers, those assessing outcomes) and how	26 (line 535-542)
	11b	If relevant, description of the similarity of interventions	N/A
Statistical methods	12a	Statistical methods used to compare groups for primary and secondary outcomes	27 (line 557)
	12b	Methods for additional analyses, such as subgroup analyses and adjusted analyses	N/A
**Results**			
Participant flow (a diagram is strongly recommended)	13a	For each group, the numbers of participants who were randomly assigned, received intended treatment, and were analysed for the primary outcome	8 (line 176-180)<br>Figure 1
	13b	For each group, losses and exclusions after randomisation, together with reasons	Figure 1

Recruitment	14a	Dates defining the periods of recruitment and follow-up	8 (line 176)
	14b	Why the trial ended or was stopped	9 (line 184-185)
Baseline data	15	A table showing baseline demographic and clinical characteristics for each group	Table 1
Numbers analysed	16	For each group, number of participants (denominator) included in each analysis and whether the analysis was by original assigned groups	Tables 2, S1, S2, S3
Outcomes and estimation	17a	For each primary and secondary outcome, results for each group, and the estimated effect size and its precision (such as 95% confidence interval)	Tables 2, S1, S2, S3
	17b	For binary outcomes, presentation of both absolute and relative effect sizes is recommended	N/A
Ancillary analyses	18	Results of any other analyses performed, including subgroup analyses and adjusted analyses, distinguishing pre-specified from exploratory	Figure S2
Harms	19	All important harms or unintended effects in each group (for specific guidance see CONSORT for harms)	Table 2
**Discussion**			
Limitations	20	Trial limitations, addressing sources of potential bias, imprecision, and, if relevant, multiplicity of analyses	15-16 (line 341-345)
Generalisability	21	Generalisability (external validity, applicability) of the trial findings	16 (line 348-354)

Interpretation	22	Interpretation consistent with results, balancing benefits and harms, and considering other relevant evidence	14 (line 301-340)
**Other information**			
Registration	23	Registration number and name of trial registry	2 (line 32)<br>5 (line 106)
Protocol	24	Where the full trial protocol can be accessed, if available	N/A
Funding	25	Sources of funding and other support (such as supply of drugs), role of funders	1 (line 23-27)

*We strongly recommend reading this statement in conjunction with the CONSORT 2010 Explanation and Elaboration for important clarifications on all the items. If relevant, we also recommend reading CONSORT extensions for cluster randomised trials, non-inferiority and equivalence trials, non-pharmacological treatments, herbal interventions, and pragmatic trials. Additional extensions are forthcoming: for those and for up to date references relevant to this checklist, see www.consort-statement.org.
